# Review and Assessment of Intellectual Property Policy Implementation in Tanzanian Universities and Research Institutions of Health and Sciences

**DOI:** 10.24248/eahrj.v6i1.678

**Published:** 2022-07

**Authors:** Kijakazi Obed Mashoto

**Affiliations:** National Institute for Medical Research

## Abstract

**Background:**

Intellectual Property Policy is one of the tools that can be used to address challenges faced by universities and research institutions in protecting and commercialising of products resulting from research activities.

**Objectives:**

The aim of this study was to the review and assess the implementation of IP policies in universities and research institutions of health and allied sciences in Tanzania.

**Methods:**

This study targeted universities and research institutions of health sciences in Tanzania. Data was collected through in-depth interviews and review of intellectual property policy documents.

**Results:**

Interviewed key informants indicated sub-optimal or lack of implementation of intellectual property policies in their respective institutions. Major reasons for lack or suboptimal implementation of intellectual property policy included limited awareness on existing institutions’ intellectual property policy, and in some institutions, lack of guidelines and regulations for implementation of intellectual property policy, and not knowing how and the importance of protecting and exploiting intellectual property.

**Conclusion:**

Sub optimal and non-implementation of Intellectual Property policy in the studied institutions can be partly attributed to lack of policy guidelines and low awareness on intellectual property policy among staff members. Effective approaches for dissemination of approved Intellectual Property policy, regulations and guidelines will enhance its implementation and hence promote IP protection and commercialisation.

## BACKGROUND

Recent national and international developments in intellectual property treaties, legislation, and frameworks are having profound effects on innovation systems and on how public and private research and development institutions implement their missions and how health and agricultural innovations reach the poor.^1^ Research institutions and universities have assumed an expanded role in science and technologies by venturing into commercialisation activities of their institution's research and development.^2^ As such, they are expected to make direct contributions to economic development and the wellbeing of society. This role requires them not only to produce but also commercialise knowledge, i.e. to use research results to create Intellectual Property (IP) and contribute to new processes and products tradable in the market.^3^ However, universities and research institutions are faced with a number of challenges in generating, protecting and commercialising their IP.^4^

As the public sector devotes more of its efforts to humanitarian missions, and engages in more Product Development Partnerships in the fields of health and agriculture, there is need to critically consider the role of intellectual property in a broader innovation context. Intellectual property rights are a critical tool for fostering innovation. Managed judiciously, they balance private rights and public necessity in a manner that, overall, encourages innovation. Understanding how intellectual property fits into the much broader context of innovation and product development is important for any public sector entity.^1^

Despite efforts made by the government of Tanzanian to establish a number of Research and Development (R&D) institutions as well as training of researchers countrywide, the benefits of research have not been fully realised.^5^ Evidence indicate that there is low use of IP in Tanzania and this is associated with lack of IP policy or inadequate IP guiding policies, inadequate IP knowledge and awareness, and limited capacity for IP system.^6^ For effective translation of research results into intellectual assets, universities and research institutions need suitable policies that provide structure, mechanisms and frameworks for ownership, incentives, benefit sharing, collaborative research, commercialisation and management of publicly and privately sponsored research. Contribution of the Private Sector to Research and Development (R&D) is currently limited due to weak incentives to invest in R&D, low understanding and appreciation of the financial and economic advantages of adopting new technologies, and weak multi-stakeholder platforms and partnerships.^7^

An IP policy is a formally-adopted document which clarifies the ownership of and right to use the IP resulting from the institution's own or collaborative R&D activities. IP policy sets out the rules of the institution on how to accurately identify, evaluate, protect and manage IP for its further development, usually through some form of commercialisation. IP policy provides a transparent framework for cooperation with third parties and provides guidelines on the sharing of economic benefits arising from the commercialisation of IP.^4^

Recent assessment conducted by the Ministry of Education, Science and Technology, and a pilot study conducted by Tanzania Commission for Science and Technology (COSTECH) in collaboration with National Bureau of Statistics (NBS) indicated that the use of IP system by universities of health and allied sciences, and health research institutions is very low (unpublished results). Numerous previous studies revealed that challenges associated with the low use of IP system by universities and research institutions include; limited capacity for IP system and lack of IP policies and guidelines.^8,9^ The aim of this study was to review and assess implementation of IP policy in universities and research institutions of health and allied sciences in Tanzania. This study generated evidence on challenges in implementing IP policies in universities and health research institutions in Tanzania.

## METHODS

The study targeted universities and research institutions of health sciences in Tanzania. Data was collected through in-depth interviews and reviews of intellectual property policy documents.

The Ministry of Health, Community Development, Gender, Elderly and Children (MOHCDGEC) through the National Institute for Medical Research (NIMR) in Tanzania was in the process of developing IP policy for the health sector. The IP policy drafting team is composed of focal personnel from 13 research institutions and universities. Hence, request to share intellectual property policy for review was sent to 13 institutions. (Table 1)

**TABLE 1:** Existing and Reviewed Intellectual Property Policy Documents, and Interviewed Institutions

Name of Institution	IP Policy	Reviewed IP Policy	In-depth interview conducted
National Institute for Medical Research (NIMR)^*^	√	√	
Muhimbili University of Health and Allied Sciences (MUHAS)	√	√	√
Sokoine University of Agriculture (SUA)	√	√	
University of Dar es Salaam (UDSM) – Mbeya College of Health and Allied Sciences (MCHAS)	√		√
Ifakara Health Institute (IHI)	√	√	√
Kilimanjaro Christian Medical College (KCMco)	√		
University of Dodoma (UDOM)	√		
Hubert Kairuki Memorial University (HKMU)			√
Nelson Mandela African Institution of Science and Technology (NM-AIST)	√		√
Tanzania Food and Nutrition Centre (TFNC), Open University of Tanzania (OUT)	√	√	√
Aga Khan University in Tanzania (AKU)			√
Kampala International University in Tanzania (KIUT)			√
**Statistics**	**9/13 = 69.2%**	**5/9 = 55.5%**	**8/13 = 61.5%**

* IP policy final draft await approval

Face to face in-depth interviews were conducted using Kiswahili language with 8 key informants (Directors of research and publications) from Tanzania Food and Nutrition Centre (TFNC), Hubert Kairuki Memorial University (HKMU), University of Dar es Salaam (UDSM), Ifakara Health Institute (IHI), Kampala International University in Tanznaia (KIUT), Agha Khan University (AKU) in Tanzania, Muhimbili University of Health and Allied Sciences (MUHAS) and Open University of Tanzania (OUT). An In-Depth Interview (IDIs) guide was developed and pre-tested amongst NIMR researchers. These researchers were not included in the actual interview. In-Depth Interview (IDI) topics included; implementation of IP policy and challenges associated with protection of intellectual properties created through research activities. Trained research assistants conducted all interviews in privacy at the workplace, using the developed interview guide. The main questions in the - guide were followed by a probing set of questions according to obtained responses. IDIs with key informants were audio-recorded and lasted between 20 and 30 minutes. The information collected was based on the principles of theoretical saturation.^10^ Data was collected between April and May 2021.

### Data Analysis

Kvale^10^ loosely guided the content analysis approach used for analysing the qualitative data. The author transcribed the audio-records verbatim and coded all transcripts on the margin of each transcript. The codes were sorted manually into categories. Quotes that were used to illustrate participants' views are reflected in this paper. Universities and research institutions were assigned codes which were used to distinguish them. This was done to ensure anonymity and to protect participants’ identity.

### Ethical Consideration

The study was granted ethical approval waiver from the Medical Research Coordination Committee (MRCC), Ref number NIMR/HQ/R.8a/Vol II of 2020/122 Data was collected for a needs assessment study aimed at generating information to inform IP policy developing process for universities and research institutions.

### Participants Description

Two institutions responded to the request by sharing their IP policy documents through email.^11,12^ Three institutions' IP policy documents^13,14,15^ were accessed via internet search. Four institutions, namely; TFNC, Agha Khan University, HKMU and KIUT did not have standalone IP policy documents (Table 1). Four institutions, namely; UDOM, UDSM, KCMUco and NM-AIST have IP policy in place, however, efforts to get their policy documents did not bear fruits, and hence after several reminders, decision was made to exclude them from the study. Therefore, 5 IP policy documents were reviewed (Table 1).

## RESULTS

### Findings of IP Policy Documents Review

While IHI IP policy document did not have vision and mission, NIMR's IP policy vision lacked focus on safe guarding the interest of the institute and those involved in IP generation, protection and commercialisation of intellectual properties. All reviewed IP policy documents had organisational structure and procedures through which documents, publications, inventions and discoveries made in the course of research and other activities are identified, protected and are made available to the public through channels of commerce. However, NIMR and IHI IP policy documents had inadequate mechanisms for determining IP ownership.

There are variations in the way institutions set up their IP management offices. In Sokoine University of Agriculture (SUA), the Directorate of Postgraduate Studies, Research, Technology Transfer and Consultancy is responsible for managing IP matters at the University, and the Deputy Vice Chancellor (Academic) provides the oversight role in the implementation of the IP policy. For IHI, creator/inventor/scientist disclose their IP interests to the Director who reports all collected disclosures to the board of trustees. Similar set up have been observed in 3 institutions; OUT, MUHAS and NIMR. In these institutions, the IP Unit, under the Directorate of Research and Publications (DRP)/Directorate of Research Coordination and Promotion is responsible for the day-to-day administration of IP related activities. However, the Intellectual Property Management Committee (IPMC) decides on what to protect and the modality of commercialising specific Intellectual Property Rights (IPR). For Open University of Tanzania and MUHAS, the Committee is chaired by the Director of Research and Publications. For NIMR, the Committee reports to the Director General, who is the Chairperson of the same Committee. However, in all the 3 institutions (OUT, NIMR and MUHAS), the IPMC is constituted by the Legal Officer, appointed academic members from the Schools/Institutes/Directorates/Colleges, undergraduate and postgraduate student representatives and IP technical expert.

The rights of indigenous and traditional medicine knowledge holders have been mentioned in IHI and SUA IP policy documents. IHI and SUA considered protection of indigenous knowledge holders from any infringement of their rights, misappropriation, and misuse or exploitation of their knowledge. However, clauses of rewarding indigenous knowledge holders are only included in IHI's IP policy document.

Apart from publications of research results which are normally used to promote scientific and academic staff, there is no other non-monetary incentives mentioned in the reviewed universities' IP policy documents. Although in the NIMR IP policy draft there is mentioning of using research outputs as a major criterion for promotion of scientific staff, it is not clear on how much weight is assigned to different research outputs such as publication of research results, submitted application for IPRs, registered research products, granted patent or trademarks and commercialised IP.

While 4 IP policy documents of MUHAS, SUA, OUT and NIMR, determine institution's ownership of IP based on the significant use of the institution's resources, IHI IP policy document indicated inventor/employee ownership. However, definition of significant use of resources varies between institutions and there is no definition of significant use in the SUA and NIMR IP policy document. For OUT, utilising of 35% of the university's resources for creation of IP is considered significant use. For MUHAS, resources use is categorised into moderate and/or significant use. Moderate university's resource contribution includes; use of office space, library, IT services and University name. Significant university's resource contribution includes; use of University finances for IP development, protection or commercialisation; use of University's account system for grant management; engagement of University laboratory staff and use of laboratory equipment. For copyrightable materials, if the institution's contribution is considered moderate, the IP creator becomes the owner. However, the institution retains the perpetual non-exclusive and irrevocable rights to non-commercial reproduction and distribution of the copyright materials for teaching and research. The creator retains 100% ownership of the IP developed outside of the institution and without significant use of the institution's resources. Conditions for co-ownership apply in cases where IPR are obtained after the creator is no longer employed by MUHAS, provided the creation steps happened when he/she was still an employee of MUHAS. For co-owned IPR between the creator(s) and the University, the creator cannot assign or license the IPR (copyrights, patents, trademarks etc.) without the written consent of the University.

In addition to university/institution and inventor ownership, all IP policy documents recognise co-ownership. The rights of students to own IP arising from their research works is recognised by OUT, MUHAS, SUA and NIMR IP Policy documents. The student owns copyright to their scholarly work subject to royalty free license to the University/Institute to reproduce and publish. However, ownership of any other IP that the student create or discover in the course of their research is governed by the terms in the contractual agreement in cases where; (i) the student has made significant use of the University resources (such as facilities or equipment), or (ii) the student received financial support from the University or another sponsor in the form of wages, salary, stipend or grant funds for the research, or (iii) the research is subject to materials transfer agreement, confidential disclosure agreement or other legal obligations that restricts ownership of the IP. For MUHAS and OUT IP Policy documents, in the absence of such terms, conditions for co-ownership between student and University as stipulated in the IP policy applies. IHI IP policy document is silent about student IP ownership.

While NIMR and MUHAS IP policy documents do not describe how ownership of IP created by visiting researchers will be determined. IP policy of OUT requires visiting researchers to transfer to the University any intellectual property they create in the course of their activities arising from their association with the University. Such individuals are treated as if they are part of OUT employees.

For both SUA and OUT, the rights related to intellectual property that is created during an academic visit by the employee of OUT or SUA to another university is governed by an agreement between the employee's University and such other university. If the IP Rights of the employee's University are not affected, the IP created during the visit shall belong to the other university unless otherwise provided in an agreement. Intellectual property created through commissioned work by a consultant belongs to the University/Institute, unless otherwise provided by written agreement between that person and the University/Institute or the third party. This policy is similar across all reviewed IP policy documents.

For MUHAS, SUA, OUT, IHI and NIMR, ownership of any IP that is made, discovered or created in the course of research funded by a private sponsor, the ownership is governed by the terms stipulated in the relevant agreements such as; grant or research agreement, materials transfer agreement, confidential disclosure agreement or other legal obligation affecting ownership. In addition to above mentioned agreements, NIMR IP policy document has a Memorandum of Understanding (MoU) for the same. However, the later document is not legally bound to settle ownership of created IP. In the absence of an agreement, institution ownership is claimed.

IP policy documents of IHI, NIMR, MUHAS and OUT are silent about IP ownership by non-employees who are neither visiting researchers nor students but associate with the institute/university in the creation of IP, in most cases, these are holders of traditional medicine knowledge or indigenous knowledge. SUA IP policy document require such persons to transfer to the University any intellectual property they may have created in the course of their activities arising from their association with the University. Such individuals will be treated as if they were employees of SUA.

In all the 5 institutions, intellectual property created through collaboration of two or more institutions, terms and conditions of intellectual property rights in the collaborative research contract is used to determine share of ownership.

MUHAS IP policy document explicitly described the university's position on open access, open innovation, publication and collaboration. After completion of research, data on which the research work was based is made available to other members of the university for royalty-free non-commercial use for teaching and research activities. Notwithstanding the above, members of the university have the collegial obligation to allow the owners(s) of such data a first opportunity to exploit that data for publishing. After its publication in the open literature, data on which research work is based on is made available for royalty-free non-commercial use by anyone who requests it. The data must bear the appropriate copyright marks. Exceptions to these rules are allowed only when the research is subject to; confidentiality requirements due to contractual arrangements with a sponsoring agency, delays associated with patent applications, or to university policy constraints on research involving human subjects or animals. In the case of contractual limitations, all collaborators must be made aware of, and agree in advance to such constraints. In OUT IP policy document, there are provisions for University and researcher to jointly own data generated through research activities and either party have the right to access and use the data for research purposes. Sponsors of research may own the collected data for research for purpose. Collaborators also have unrestricted access to all data obtained or collected through collaborative research activities. In spite of these provisions, entitlement to the ownership of primary data, software, and other products of research may vary, depending on the circumstances under which the research is conducted. As such, ownership of data would be specified in the contract agreement to be signed by the two or more parties.

Practice of sharing income arising from commercialisation of IP is based on the net value arrived (after deducting all applicable overheads that went into IP development, protection or commercialisation). For SUA, IHI and MUHAS, revenues accrued from commercialisation of IP are equally shared between the creator and institute or university unless legal requirements or contractual agreement dictates otherwise. However, in cases where significant or moderate institution's resources were utilised, such as use of University finances in product development, filing for patents and business incubation, the payment of royalties to MUHAS become higher than 50% as agreed between the creator(s) of IP and University. Except for SUA's IP policy document, all other reviewed IP policy documents have provisions on how the share of the institution's royalties is distributed between departments and units (Table 2).

**TABLE 2:** Distribution of Institution's Royalty Share

	NIMR	MUHAS	IHI	OUT^a^	SUA
Administration	20%	20%	10%	–	Not stated
DRP/DRCP/DG office	20%^*^	15%	10%	30% or 25% or 20%	Not stated
School/Lab/Innovation hub^**^	10%	5%	20%	–	Not stated
Department/TT Desk^***^	10%	10%	10%	30% or 25% or 20%	Not stated
**Total institution's share**	**60%**	**50%**	**50%**	**60% or 50% or 40%**	**50%**

* The 20% is equally distributed between DRCP and administration office at NIMR Headquarters

** School or Institute for MUHAS, Innovator's laboratory for NIMR and Innovation Hub for IHI

*** Innovator's department for NIMR and MUHAS, Technology Transfer Desk for IHI

a Division of income based on the types of intellectual property rights and the level of income. For patented inventions or discoveries with the level of income of USD 20,000 of IP royalties, University get 40%. Patented inventions with the level of income of over USD 20,000 of IP royalties, University gets50%. For other types of intellectual property rights (not patent) if IP royalty is USD 5,000, university get 40%, and if over USD 5,000, university gets50%.

For OUT, division of income is based on the type of Intellectual Property Rights (IPR) and the level of income. For patented inventions or discoveries with level of income of USD 20,000 of IP royalties, the inventor gets 60% and the University 40%. Patented inventions with the level of income of over USD 20,000 of IP royalties, the inventor gets 50% and the University 50%. Division of income derived from IP other than patents, for IP income of the first USD 5,000 of IP royalties, the inventor(s) get 60% and university 40%, and income of over USD 5,000 of IP royalties, the inventor(s) get 50% and university 50%. In both cases, University share is equally distributed between the inventor's department and the directorate of research and publication (Table 2).

For IHI, the apportionment due to the creators attract eligibility of all parties including local and indigenous communities who have collaborated in one way or the other and is shared equally between parties unless provided otherwise by legal requirements and/or contractual obligations. A community is treated as one single party unless community participation is by means of bona fide legal persons. In the absence of an agreement, multiple inventors receive equal portion of the inventors' share of net revenue. Where multiple inventors are located in different units, the unit of leading innovator will receive the total share of the net revenue. For NIMR, joint creators or inventors decide on how the 40% share is distributed among themselves.

Similar policy of assigning the IPR back to the creator in the event the institute or university is not interested in exploiting the created IP have been observed in the reviewed documents. NIMR may, in writing, allow the individual researcher to claim ownership while retaining its worldwide royalty-free licence to use the said intellectual property rights. Similarly, MUHAS assign the IPR back to the creator in writing. However, in case of successful future venture of the IP outside of MUHAS, the university receive its share of royalties as agreed between the creator and University in the IP Revenue Sharing Agreement. Vice versa, creators of IP to which MUHAS has no ownership may elect to assign IP to be managed by the university upon mutual agreement, provided that there is no conflict with the co-creators, sponsors, third party or applicable laws and regulations. For OUT, inventor(s) receive notification at least one month prior to any act or any intentional omission liable to prevent the obtainment of protection. In such cases the inventor(s) have the option to acquire related IP Rights. IP policy document of SUA have no provision regarding what happen in the event the university decides not to exploit the created IP.

## FINDINGS OF KEY INFORMANT INTERVIEWS

Under the central theme of ‘implementation of IP policy', 4 categories emerged (Table 3). The first category describes implementation of available policies and guidelines to manage IP. The second category underscores the performance of policies, regulations and guidelines in protection and commercialisation of IP. The third category describes how IP awareness impacts the implementation of IP policy and guidelines. The forth category addresses feasibility of improving existing IP policy or adopting a model IP policy.

**TABLE 3:** Emerged Categories of Intellectual Property Policy Implementation Theme

Themes	Categories
Implementation of intellectual property policy	Implementation of availablepolicies and guidelines to manage IP Performance of policies, regulations and guidelines in protection and commercialization of IP Intellectual property awareness as a barrier to IP policy implementation Willingness to adopt or improve existing IP policy

### Implementation of Available Policies, Regulations and Guidelines to Manage IP

This study observed that IP policy, regulations and guidelines are either lacking or inadequately implemented in universities and research institutions of health and sciences in Tanzania. This is illustrated by the following quotes from key informants:

*“Legal mandate requires the institute to conduct research, but the law that established our institute have not been effectively enforced due to lack of implementation guidelines. Now, we have developed the guidelines and we have submitted the documents to relevant authorities for approval”*

(Male respondent from Institution 6).

“*There is no means of identifying and evaluating findings with commercialisation potential. If a student or staff invents today, or come up with an innovation, we will face some challenges as there are no guidelines on how to go about protecting and commercialising the invention”*

(Male respondent from Institution 8).

“*Sometimes we encounter challenges when partners ask how do we protect the interest of our scientists and researchers who come up with innovations or inventions, as the policy we have is too general and does not adequately address issues of ownership”*

(Female respondent from Institution 2).

*“I cannot say that we have specific mechanisms for identifying innovations or research with potential for commercialisation because we do not have an IP policy in place. The available research policy has been approved last year and it does not cover much on IP related issues”*

(Male respondent from Institution 6).

Similar to the findings from IP document reviews, key informants emphasised on the importance of having IP policy in order to safe guide institution's and staff's interests.

“*Having the IP policy helps us to raise staff's and stakeholders’ IP awareness and provide guidance for protection of the IP they create”*

(Male respondent from Institution 1).

*“We live by the slogan which says “Protect before you project”, that is the slogan we use to advice researchers and students to be keen in protecting their IP/innovations*

(Male respondents from Institution 3).

*“When there is no IP policy, there is a possibility that the institute will be robbed off it's IP. For example, 30 years ago, we were involved in a joint program in one of the region. The program was externally funded. We developed malnutrition conceptual framework but as we speak, nobody knows that we were actively involved in developing that framework. All the credits went to the funder who claimed the ownership of the conceptual framework. May be if we had an IP policy in place, the situation would have been different”*

(Male respondent, Institution 6).

### Intellectual Property Awareness as a Barrier to IP Policy Implementation

Inadequate implementation of IP policy is linked with lack or limited IP awareness, knowledge and capacity.

*“Our IP policy is being implemented, however, more IP trainings and awareness campaigns are needed to empower our students and employees to protect their innovations and research findings”*

(Male respondent from Institution 3).

*Most of our staff and students have limited knowledge on the applications of IP policy, so it is important to widely disseminate the policy and raise employees' and students' awareness on the existing policy*

(Male respondent from Institution 5).

*“The policy is still at its infant stage and thus we have not encountered any challenges in its implementation. All is needed now is to continue raising awareness on the use of IP policy”*

(Male respondent from Institution 1).

### Performance of Policies, Regulations and Guidelines in Protection and Commercialisation of IP

All key informants reported that the mission and orientation of universities and research institutions is driven by research and academic, and that social and economic development are the priorities of such institutions.

“*We do not have any research product which we have commercialised so far. We are more oriented to service provision. We use research results to inform teaching and health practices. So I would say that we have indirectly contributed to the social and economic development in this country. Improved education leads to reduced illiterate individualsand improved health practices results in improved health status and increased involvement in income generation activities and productivity”*

(Male respondent, Institution 4)

*“Our institute's orientation is towards serving the public, and hence SMEs are being trained on preparation of various food formula and Ready to Use Therapeutic Food (RUTF). We conduct research to improve access to nutrition services to the public. We train SMEs to produce and make it easy for the public to access our products. But, the institute does not commercialise any product it creates”*

(Male respondent from Institution 6).

Surprisingly, certification was the most frequently mentioned type of IPR commercialisation but none of the key informants mentioned that their respective institutions had been granted certification rights for any of their research products, services or processes. Three institutions (SUA, MUHAS and UDSM) had been granted patents (Table 4). Apart from copyrights and patents, other types of IPR were not common as narrated by key informants.

**TABLE 4:** Intellectual Property Policy Implementation Status and Types of Granted Intellectual Property Rights

	NIMR	MUHAS	IHI	OUT	SUA	UDSM	KIUT	AKU
**Granted intellectual property rights**								
Patents	0	4	0	0	11	3	0	0
Trademarks	0	0	0	0	0	0	0	0
Copyrights	Yes	Yes	Yes	Yes	Yes	Yes	Yes	Yes
Certifications	0	0	0	0	0	0	0	0
Trade secrets	Yes	Yes	Yes	0	Yes	Yes	0	0
PBRs	NA	NA	NA	NA	3	0	NA	NA
**IP Policy**								
Availability	Draft	Yes	Yes	Yes	Yes	Yes	No	No
Implementation status	No	Yes	No	No	Yes	Yes	No	No

PBRs - Plant Breeders’ Rights; NA - not applicable as new plant variety is not the focus of the institution.

*“So far we have about 3 to 4 patents for our products, one of them is the herb based antimicrobial substance, and another one is the nutritional supplement which is already in the market. The other IP is in its very early stage and therefore I cannot disclose its information now”*

(Male respondent from Institution 1).

*The University has IP policy but we have not register any of the research findings or innovation or invention for patent and other types of IPRs apart from copyrights. This is because of limited knowledge on IP protection and commercialisation, the types of research we conduct do not translate into tradable IP and limited knowledge on the use of IP policy by our students and employees”*

(Male respondent from Institution 5).

*Having IP policy enabled us to register many of our IP for patent and copyrights. So far, 3 patents have been granted. Over 30 IPs have been submitted to BRELA for registration and application for patents and copyrights*

(Male respondent from Institution 1)

### Willingness to Adopt or Improve Existing IP Policy

When asked if their respective institutions will be willing to adopt a model IP policy or revise their existing IP policy documents to address gaps identified by the review performed by this study, key informants responded as follows:

*“We are looking forward to the operationalization of the National IP policy so that we ensure that our institution's policy is aligned it. Having a National IP policy is important as institutions will be guided to developing their IP policies that aligns with that of the nation, but for now, what you have started (NIMR), the coordination of developing a model IP policy for universities is so important”*

(Male respondent from Institution 1).

*“Well, now that you ask me about IP policy, I think it is a good idea to have one. I will share this idea with postgraduate team and then see the possibility of developing IP policy*”

(Male research respondent from Institution 7).

*“The move to involve universities and research institutions in developing a model IP policy is a right one, it will enable scientists and researchers to have one voice and power to stand for their rights when they create IP using own, public or private sources of funds, or create IP in collaborations and partnerships”*

(Female respondent from Institution 2).

*“I real wish that something is done to fasten the approval of the National IP policy, this will help people, particularly those in medical schools (staff and students) know their rights for what they create”*

(Male respondent from Institution 8).

## DISCUSSION

Intellectual property in its broadest form is the manifestation of ideas, creativity and invention in a tangible form. IP in the broad sense underpins all of the activity of a university and research institutions. However, many researchers make the assumption that intellectual property means primarily patents, and therefore think that other forms of intellectual property rights are of no direct relevance to them. The common type of IPRs used by the studied institutions is copyrights, and none of the institution used trademark for protection of the institutions' IPs. Out of 8 institutes, 5 confirmed that they used trade secrets for protection of their products or research outputs.

Trademarks are a form of IP protection that serves to distinguish the products or services of one individual, company, or organisation from the products or services of the others. A trademark can be a word, phrase, symbol, design, or a combination thereof. Trademarks can even be sounds or colours, if they are in some way distinctive, that create an immediate association in the mind of the consumer between the trademark and the good. IP protection for a trademark confers an exclusive right to use the mark in commerce. Evidence from this study indicates that trademarks are overlooked and undervalued form of intellectual property. Perhaps, research institutions and universities in Tanzania are not aware of complementing protection provided by trademarks to other forms of statutory IP protection.^1^

Advocating for one IP model may not be appropriate as there is a wide range of institutional types, with different strengths and different objectives, and ultimately different business models. However, the reviewed IP policies and the institutions' set up for management of IP did not exhibit significance differences. Thus, a strategy needs to be directed to “best fit” the objectives and/or business model of the institution.^1^

Like public universities and research institution in Tanzania, most of the institutions in the United States of America (USA) follow the university ownership model where there is a crucial role of technology transfer office to commercialise the IP generated. Sweden has an inventor ownership model where the inventor has freedom to work on his/her patent for its commercialisation.^16^ The revenue sharing mechanism could be of linear (fixed) and non-linear (variable) types. In the linear mechanism, there would be a fixed share of revenue distribution among those who contributed in the IP generation process, whereas, in the non-linear mechanism, revenue is distributed based on milestone payments after achieving the pre-set target amount during commercialisation/marketing. Most of the European and Australian universities follow this type, and the same is being practised in Tanzania. However, there is some similarity between OUT and Boston college where revenue share of the inventor after licensing is of non-linear, step-down type, where by up to $5000 IP income, the inventor share is 100%, from $5001 to $10,000, inventor share is 85%, and from $10,001 and above, inventor share is 50% and the rest goes to the provost.^17^

Any new or revised IP policy (and IP strategy) will have to be “sold” to people both inside and outside an institution. It is important to explain *what* the policy contains and *why* the policy is designed the way it is. And perhaps staff at multiple levels should be involved in developing and revising, as needed, the IP policy. This group will be able to have extensive discussions about the role and function of intellectual property in the organisation. These discussions will be an effective mechanism for building capacity and staff support of the policy. Some of the most controversial issues can be resolved before they become an obstacle.^1^

Intellectual property is a tool to foster innovation and an instrument to achieve humanitarian objectives. Since research activities may result in tradable IP which can therefore be owned and sold, university and research communities should be encouraged to invest, based on the profit potential from research activities. IP protections can prevent access by some individuals and populations. However, there are many ways for intellectual property to be distributed, utilised and put to work for the interests of the public. Hence there is no need to either fear intellectual property or embrace it blindly, it should be managed to maximise the benefits of research for all of society, especially the poor.^1^

Policies to promote the creation and management of intellectual property by research institutions and universities in Tanzania should give first priority to advancing the mission of those institutions. This means, technology transfer should support the larger mission and not merely be seen as potential revenue sources.^1^

### Limitations of the study

Relying on the empty promises made by key informants from some of the institutions that they will send their IP policy documents via email was an obstacle to this study. However, it is possible that the gaps identified in the reviewed documents do not differ with what might have been identified in the missed IP documents, because high learning institutions have more or less similar goals which are normally aligned with the IP policy goal.

## CONCLUSION

Sub optimal and non-implementation of IP policy in the studied institutions can be partly attributed to lack of policy guidelines and low IP policy awareness among staff members. Effective approaches for dissemination of approved IP policies and their guidelines will enhance its implementation and hence promote IP protection and commercialisation. There is also a need to put in place mechanisms for protection of rights of traditional medicine knowledge holders.
